# Optimal Information Transfer and the Uniform Measure over Probability Space

**DOI:** 10.3390/e25060875

**Published:** 2023-05-30

**Authors:** William K. Wootters

**Affiliations:** Department of Physics, Williams College, Williamstown, MA 01267, USA; william.wootters@williams.edu

**Keywords:** information transfer, quantum reconstruction

## Abstract

For a quantum system with a *d*-dimensional Hilbert space, suppose a pure state |ψ〉 is subjected to a complete orthogonal measurement. The measurement effectively maps |ψ〉 to a point $\left(p_{1},\ldots ,p_{d}\right)$ in the appropriate probability simplex. It is a known fact—which depends crucially on the complex nature of the system’s Hilbert space—that if |ψ〉 is distributed uniformly over the unit sphere, then the resulting ordered set $\left(p_{1},\ldots ,p_{d}\right)$ is distributed uniformly over the probability simplex; that is, the resulting measure on the simplex is proportional to $dp_{1}\cdots dp_{d-1}$. In this paper we ask whether there is some foundational significance to this uniform measure. In particular, we ask whether it is the optimal measure for the transmission of information from a preparation to a measurement in some suitably defined scenario. We identify a scenario in which this is indeed the case, but our results suggest that an underlying real-Hilbert-space structure would be needed to realize the optimization in a natural way.

## 1. Introduction

This paper brings together two questions pertaining to the structure of quantum theory. The questions concern (i) a quantum-mechanically natural measure on the probability simplex, and (ii) the optimization of the transfer of information. I now present the two questions.

(i) *A quantum-mechanically natural measure on the probability simplex*

Given a quantum system with a *d*-dimensional Hilbert space, let us suppose its state is pure but otherwise unknown, by which we mean that it is distributed *uniformly* over the unit sphere in the Hilbert space. (The uniform distribution is the unique distribution that is invariant under all unitary transformations). Now suppose we perform on the system a complete orthogonal measurement, represented by an ordered set of *d* orthogonal projection operators $\left(\Pi_{1},\ldots ,\Pi_{d}\right)$. For any given pure state |ψ〉, the measurement maps the state to an ordered set of probabilities, namely, the probabilities of the outcomes of the measurement if the state is |ψ〉. The mapping is given by

(1) $$|\psi \rangle \to \left(p_{1},\ldots ,p_{d}\right)=\left(\langle \psi \right|\Pi_{1}\left|\psi \rangle ,\ldots ,\langle \psi \right|\Pi_{d}|\psi \rangle ).$$

The ordered set $\left(p_{1},\ldots ,p_{d}\right)$ resides in the (d−1)-dimensional simplex $\Sigma_{d}$ defined by the conditions $p_{j}\geq 0$ and $p_{1}+\cdots +p_{d}=1$. Now, if our knowledge of |ψ〉 is characterized by the uniform distribution over the unit sphere, what distribution, or measure, on the simplex $\Sigma_{d}$ characterizes our knowledge of the probabilities $\left(p_{1},\ldots ,p_{d}\right)$? This question was answered in 1974 by Sýkora [1]. It turns out that the resulting measure is the *uniform* measure. That is, if we parameterize the points of the simplex by the first d−1 probabilities $p_{1},\ldots ,p_{d-1}$, the induced measure dμ is given by

(2) $$d\mu =\left(d-1\right)!\;dp_{1}\ldots dp_{d-1},$$

which is normalized in the sense that

(3) $$\int_{\Sigma_{d}}d\mu =1.$$

For example, if d=2, we start with a random state uniformly distributed over the surface of the Bloch sphere. Suppose we perform on this state the orthogonal measurement whose first and second outcomes are represented respectively by the north and south poles of the sphere. Then the probability $p_{1}$ of the first outcome is $p_{1}=\frac{1}{2}\left(1+\cos\theta \right)$, where θ is the angle between the north pole and the point representing the state being measured. The area element on the sphere can be written as d(cosθ)dϕ, and the range of the azimuthal angle ϕ is independent of θ; so for our random state, the quantity cosθ is distributed uniformly between −1 and +1. It follows that $p_{1}$ is distributed uniformly over the interval $0\leq p_{1}\leq 1$.

The uniformity of the distribution over the probability simplex is by no means a trivial or obviously foreordained result. If quantum theory had been based on a real Hilbert space instead of a complex Hilbert space, the uniform measure on the set of pure states would have produced a rather non-uniform measure on the probability simplex. Specifically, we would have obtained the normalized measure dν given by [2]

(4) $$d\nu =\frac{\Gamma (d/2)}{\pi^{d/2}}\cdot \frac{1}{\sqrt{p_{1}\cdots p_{d}}}dp_{1}\ldots dp_{d-1},$$

which favors values of $\left(p_{1},\ldots ,p_{n}\right)$ that are close to the edge of the simplex, where one or more of the $p_{j}$s is small. The simplicity of the actual result (2) raises the following question: does the uniformity of this quantum-mechanically natural measure over the probability simplex hold any deep significance regarding the structure of quantum theory? Is there a fundamental principle implying that quantum theory *must* give rise to this measure? If so, then that principle would, for example, constitute a way of understanding why quantum theory is based on a complex rather than a real Hilbert space. (It might, for example, substitute for the principle of local tomography, which has frequently been used in reconstruction programs to rule out the real-vector-space theory [3,4]).

(ii) *The optimization of the transfer of information*

Under unitary evolution in quantum theory, information is conveyed perfectly from the past to the future. A pure state remains pure, and a mixed state does not change its entropy. In this sense, nature at a fundamental level does not waste information.

However, our actual experience of the quantum world comes to us by way of measurements, for which the appearance of a definite outcome is not described unitarily. Rather, it is described probabilistically. (This is the case even if one regards the underlying dynamics to be unitary, as in the Everett interpretation). The outcome of a measurement does not give us complete information about the prior state of the measured system, and the outcome itself constitutes novel information that was not present earlier. These facts suggest the following question: in a description of quantum phenomena that includes probabilistic measurements, is there any sense in which information is conveyed optimally from the past to the future?

### Relation between the Two Questions

I have posed versions of the information-optimization question in previous papers [2,5]. In the statements of the question I have considered, the problem boils down to determining the optimal form of the measure on the probability simplex $\Sigma_{d}$ induced by the uniform distribution over the pure states. The main upshot of my earlier work on this subject—which is consistent with the work of others [6,7,8,9]—has been that the measure that optimizes the transfer of information is the one given in Equation (4), not the one given in Equation (2). That is, if quantum theory were based on a *real* vector space rather than a complex vector space, there would indeed be a sense in which information is conveyed optimally. However, this intriguing result is spoiled by the fact that actual probability amplitudes are complex.

In the simplest example of optimal information transfer in a real-amplitude context, we imagine a beam of photons emerging from a linearly polarizing filter and then being subjected to a horizontal-vs-vertical polarization measurement. (This counts as a real-Hilbert-space example because the *linear* polarizations can be represented in such a space). A person stationed at the measuring site can gain information about, say, the probability $p_{v}$ of the vertical outcome by counting how many photons yield the vertical and horizontal outcomes. However, as long as the number of photons is finite, the amount of information gained in this way is limited. Not surprisingly, the amount of information gained depends on the prior measure one assumes for the value of $p_{v}$, and it turns out that in a suitable limit as the number of photons goes to infinity, the information is maximized if this prior measure is taken to be

(5) $$d\nu =\frac{1}{\pi \sqrt{p_{v}\left(1-p_{v}\right)}}dp_{v},$$

which is the special case of Equation (4) appropriate for d=2.

It is not hard to see that the measure (5) is the one the experimenter would naturally use if she had no initial information about the orientation of the polarizing filter from which the beam of photons emerged. The probability of the vertical outcome is $p_{v}=\cos^{2}\theta$, where θ is the angle between the filter’s favored axis and the vertical axis. In the real-amplitude theory, one would naturally assume that θ is uniformly distributed over its range, which we can take to run from 0 to π. The normalized prior measure would then be

(6) $$\frac{1}{\pi }d\theta =\frac{2}{\pi }{\left|\frac{dp_{v}}{d\theta }\right|}^{-1}dp_{v}=\frac{2}{\pi }\cdot \frac{1}{\left|2\cos\theta \;\sin\theta \right|}dp_{v}=\frac{1}{\pi \sqrt{p_{v}\left(1-p_{v}\right)}}dp_{v},$$

in agreement with Equation (5). (The factor of 2 in the first step appears because there are generically two values of θ in the interval 0≤θ≤π that yield a given value of $p_{v}$). So this is an example, in the real-amplitude context, in which the natural prior measure is also the one that maximizes the amount of information conveyed.

Our first aim in this paper is to identify a *different* optimal-information-transfer problem, ideally not too complicated, whose solution is *not* the measure given by Equation (4) but rather the one given by Equation (2), that is, the one we associate with standard, complex-amplitude quantum theory. This aim is an unusual one in a certain respect. Normally, one is given a problem and one wants to find the solution. Here, we are given a solution—the uniform measure over the probability simplex—and we are looking for a problem that has this measure as its solution. This backward-sounding procedure, however, is implicit in the question we asked earlier: in a description of quantum phenomena that includes probabilistic measurements, is there any sense in which information is conveyed optimally? We are *given* quantum theory, along with the associated measure (2) over the probability simplex, and we are looking for a problem that will serve to identify an interesting feature of this measure and hence an interesting feature of quantum theory.

We will indeed find an information-optimization problem that is solved by the measure of Equation (2). We present this problem in Section 2. In Section 3, we solve the problem and show that it leads us to the uniform measure, as promised. Then in Section 4, we ask whether we can interpret the problem within the framework of quantum theory. That is, we ask whether there is a quantum scenario in which we can see that information is conveyed optimally, without having to restrict ourselves artificially to quantum states with only real amplitudes as in the linear polarization example. We will find that there is indeed such a scenario, but it is not as straightforward as one might have liked. Finally in Section 5 we ask what insights this exercise gives us into the structure of quantum theory.

The problem we are about to lay out in Section 2 will be couched in terms of Shannon information—specifically the combination of Shannon entropies that defines the *mutual information*—and one might wonder whether Shannon information is the best way to quantify information in the setting we are considering. Shannon information is intimately related to asymptotic coding efficiency, which will not figure into our problem. However, Shannon information can also be thought of as the unique measure of information that satisfies certain natural assumptions having nothing directly to do with coding [10]. It is for this latter reason that we feel the measure is justified for our problem.

It is worth noting that there are close connections between mutual information, which we will be using here, and *Fisher information*, which is widely used in statistics [6,7,8,9]. For the discrete probability distribution $\left(p_{1},p_{2},\ldots ,p_{d}\right)$, regarded as parameterized by the first d−1 probabilities, the determinant of the Fisher information matrix *J* is $\det J=1/(p_{1}p_{2}\cdots p_{d}).$ It is no accident that the distribution (5) that maximizes the experimenter’s expected gain in information in our polarization example is proportional to the square root of detJ (for the case d=2, in which case *J* is simply a scalar function). In general, the distribution proportional to $\sqrt{\det J}$ is known as the Jeffreys prior [11]. As a prior distribution for Bayesian inference, it was originally motivated by invariance arguments, but it can also be shown to maximize information in certain settings [6,9]. Though we will not be explicitly concerned with Fisher information in what follows, some of the arguments in Section 3 below are similar to what can be found in these earlier papers.

Note that in identifying the uniform measure (2) with standard, complex-amplitude quantum theory, we are placing pure states and orthogonal measurements at the center of our argument: we obtain the quantum mechanical measure by starting with the unitarily invariant measure over *pure* states, and the probabilities in question are the probabilities of the outcomes of a complete *orthogonal* measurement. The centrality of pure states and complete orthogonal measurements in this paper reflects my own view that these structures are more fundamental than mixed states and generalized measurements. Mixed states and general POVM measurements can of course be obtained from their purer counterparts by embedding the system of interest in a larger system and ultimately ignoring part of this larger system.

## 2. The Problem

We now state our information-optimization problem. Though we are ultimately interested in gaining insight into quantum theory, the problem itself is not quantum mechanical. It is to be understood as an abstract communication problem.

The scenario begins with Alice, who sends a beam of *N* information-bearing particles to Bob. Bob has a measuring device that separates this beam into *d* subbeams, recording, for each particle, the subbeam into which it was sent (the record is the outcome of Bob’s measurement). The *d* subbeams are then sent on to participants named Carol-1, *…*, Carol-*d*, each of whom makes a *binary* measurement on each of the particles she receives. (In Section 4 and Section 5, we will discuss the significance of these binary measurements. For now, we simply take them as part of the statement of the problem). All the measuring devices in this problem—Bob’s device and the device of each of the Carols—generate their outputs probabilistically, and all the information that is conveyed is encoded in the probabilities of the outcomes. We can imagine Alice having control over the probabilities of Bob’s outcomes, so that Alice is conveying information to Bob. If we wish, we can imagine Alice also having control over the probabilities of Carol-*j*’s outcomes. Alternatively, though, we could imagine that it is Bob who controls Carol-*j*’s probabilities; the statement of the problem is independent of the source of the information.

Let $p_{j}$ be the probability of the *j*th outcome of Bob’s measurement, and let $n_{j}$ be the number of times this outcome occurs when Bob measures the *N* particles arriving from Alice. Bob’s goal is to estimate $p=(p_{1},\ldots ,p_{n})$ by observing $n=(n_{1},\ldots ,n_{d})$, but again, because *N* is finite, Bob’s information about p is limited. We quantify this information as the mutual information between p and n:

(7) $$I^{B}=I\left(p:n\right),$$

which is the average amount of information Bob gains about p upon observing n. (In our notation, we do not distinguish between a random variable and a particular value that the random variable might take, relying instead on the context to make clear which meaning is intended. In the expression I(p:n), the symbols p and n are understood to name random variables). This mutual information depends on Bob’s prior measure on the probability simplex $\Sigma_{d}$, which we write as ρ(p)dp, where $dp=dp_{1}\cdots dp_{d-1}$.

Let $q_{j}$ and $1-q_{j}$ be the probabilities of the two possible outcomes of Carol-*j*’s measurement, and let $m_{j}$ and $n_{j}-m_{j}$ be the numbers of occurrences of these outcomes when Carol-*j* measures the $n_{j}$ particles sent to her by Bob. The amount of information obtained by Carol-*j* is quantified by

(8) $$I^{C_{j}}=I\left(q_{j}:n_{j},m_{j}\right).$$

This mutual information depends on the function ρ(p), and it also depends on the function $\sigma (q_{j})$, which characterizes Carol-*j*’s prior measure $\sigma \left(q_{j}\right)dq_{j}$ over her one-dimensional probability simplex $\Sigma_{2}$. (We assume that the same function σ applies to each of the Carols).

We are interested in maximizing the sum of the information gained by Bob and the information gained by all the Carols. So we construct the quantity

(9) $$I=I^{B}+\sum_{j}I^{C_{j}}.$$

This quantity depends on *N*, and we would like to take the limit as *N* goes to infinity, but we need to take into account that *I* itself approaches infinity in this limit, growing as $(d-\frac{1}{2})\ln N$. So we aim to maximize the quantity $\tilde{I}$, defined by

(10) $$\tilde{I}=\lim_{N\to \infty }\left[I-(d-\frac{1}{2})\ln N\right].$$

Our problem, then, is to find the functions ρ(p) and σ(q) that maximize $\tilde{I}$.

In order for the argument in the following section to be mathematically sound, we need to assume that the functions ρ and σ are reasonably well behaved. We assume that both functions are differentiable in the interiors of their respective simplices. We also assume that the derivatives do not diverge too rapidly near the edges of the simplices. Specifically, we assume there are positive numbers α and β, with β<2, such that ${|d\sigma /dq|<\alpha /\left[q\left(1-q\right)\right]}^{\beta }$ and $|\partial \rho /\partial p_{j}|<\alpha /{\left(p_{1}p_{2}\cdots p_{d}\right)}^{\beta }$ for j=1,…,d−1. [12]. (One can imagine an alternative formulation of the problem that removes the temptation to introduce such extra assumptions: let $\rho_{N}$ and $\sigma_{N}$ be the optimal priors when there are exactly *N* particles. Then find suitable limiting measures ρ and σ as *N* approaches infinity. I expect the results would be the same as in the present paper, but I will have to leave that problem for future work).

## 3. Solving the Problem

Here we give the argument leading to the conclusion that the optimal function ρ for our problem is the constant function ρ(p)=(d−1)!. We will also find the optimal function σ.

We begin by estimating the mutual information $I^{B}=I\left(p:n\right)$; the error caused by using our estimate rather than the exact value will become negligible as *N* approaches infinity. First, although we are thinking of I(p:n) as the average amount of information Bob gains about p by observing n, the mutual information is symmetric, so that we can compute it as the average amount of information one would gain about n upon learning the value of p. This interpretation is the one suggested by the formula

(11) $$I^{B}=I\left(p:n\right)=I\left(n:p\right)=H\left(n\right)-H\left(n|p\right).$$

Here H(n) is the entropy of the prior probability distribution of n, which we label P(n), and H(n|p) is the conditional entropy of n given p. The exact formula for H(n) is

(12) $$H\left(n\right)=-\sum_{n}P\left(n\right)\ln P\left(n\right).$$

when *N* is very large, the vector n is likely to be very close to Np; so the function P(n) in that case is largely determined by the function ρ(p) and can be well approximated by

(13) $$P\left(n\right)\approx \frac{1}{N^{d-1}}\;\rho \left(n/N\right).$$

Thus we can approximate H(n) as

(14) $$H\left(n\right)\approx \left(d-1\right)\ln N-\int_{\Sigma_{d}}\rho \left(p\right)\ln\rho \left(p\right)dp.$$

Meanwhile, the formula for H(n|p) is

(15) $$H\left(n|p\right)=\int_{\Sigma_{d}}\left(-\sum_{n}P\left(n|p\right)\ln P\left(n|p\right)\right)\rho \left(p\right)dp,$$

where P(n|p) is the probability of the outcome n if the probabilities are those in p. This probability is given by a multinomial distribution, whose entropy, for large *N*, is well approximated as [13]

(16) $$-\sum_{n}P\left(n|p\right)\ln P\left(n|p\right)\approx \left(\frac{d-1}{2}\right)\ln\left(2\pi eN\right)+\frac{1}{2}\ln\left(p_{1}\cdots p_{d}\right).$$

We can therefore write

(17) $$H\left(n|p\right)\approx \left(\frac{d-1}{2}\right)\ln\left(2\pi eN\right)+\frac{1}{2}\int_{\Sigma_{d}}\rho \left(p\right)\ln\left(p_{1}\cdots p_{d}\right)dp.$$

Combining Equations (14) and (17), we have

(18) $$I^{B}=I\left(p:n\right)\approx \left(\frac{d-1}{2}\right)\ln\left(\frac{N}{2\pi e}\right)-\int_{\Sigma_{d}}\rho \left(p\right)\ln\rho \left(p\right)dp-\frac{1}{2}\int_{\Sigma_{d}}\rho \left(p\right)\ln\left(p_{1}\cdots p_{d}\right)dp.$$

Before we continue with our calculation, we pause to note that if we were simply to maximize $I^{B}$, then for very large *N* we would be maximizing the right-hand side of Equation (18). One can show that the distribution ρ(p) that achieves the maximum in this case is the one given by Equation (4), that is, the distribution associated with a real Hilbert space [2]. So, the fact that we include in Equation (9) the information transmitted by Bob to the Carols will make a crucial difference in determining what function ρ(p) emerges as optimal.

Continuing now with our calculation, we compute $I^{C_{j}}$, which we can write as

(19) $$I^{C_{j}}=I\left(q_{j}:n_{j},m_{j}\right)=H\left(n_{j},m_{j}\right)-H\left(n_{j},m_{j}|q_{j}\right).$$

The entropies on the right-hand side can be expanded via the additivity of Shannon entropy.

(20) $$\begin{array}{cc} \; & H\left(n_{j},m_{j}\right)=H\left(n_{j}\right)+H\left(m_{j}|n_{j}\right)\\ \; & H\left(n_{j},m_{j}|q_{j}\right)=H\left(n_{j}|q_{j}\right)+H\left(m_{j}|n_{j},q_{j}\right). \end{array}$$

Now, the number of particles, $n_{j}$, entering Carol-*j*’s apparatus has nothing to do with the probability $q_{j}$ of the first outcome of Carol-*j*’s measurement. So $H(n_{j}|q_{j})$ is equal to $H(n_{j})$, and those two terms cancel when we carry out the subtraction in Equation (19). Thus we have

(21) $$I^{C_{j}}=H\left(m_{j}|n_{j}\right)-H\left(m_{j}|n_{j},q_{j}\right).$$

This equation is similar in form to the Equation (11) defining $I^{B}$, but there is now an additional conditioning (on $n_{j}$). We use this similarity to find an approximate formula for $I^{C_{j}}$ analogous to our Equation (18) for $I^{B}$. The role of *N* is now played by $n_{j}$, and the role of ρ(p) is now played by $\sigma (q_{j})$. The differences are these: (i) whereas in the calculation of $I^{B}$ the number of incoming particles (*N*) was fixed, now we need to average over $n_{j}$, and (ii) Carol-*j*’s measurement has only two outcomes, so we replace *d* with 2. This gives us

(22) $$I^{C_{j}}\approx \langle \frac{1}{2}\ln\left(\frac{n_{j}}{2\pi e}\right)-\int_{0}^{1}\sigma \left(q_{j}\right)\ln\sigma \left(q_{j}\right)dq_{j}-\frac{1}{2}\int_{0}^{1}\sigma \left(q_{j}\right)\ln\left[q_{j}\left(1-q_{j}\right)\right]dq_{j}\rangle ,$$

where the angular brackets indicate an average over the prior distribution of $n_{j}$. Of the three terms inside the brackets, only the first term will need to be averaged in this way. Using the fact that for large enough *N*, the value of $n_{j}$ will likely be very close to $Np_{j}$, we can write

(23) $$\langle \ln n_{j}\rangle \approx \int_{\Sigma_{d}}\rho \left(p\right)\ln\left(Np_{j}\right)dp=\ln N+\int_{\Sigma_{d}}\rho \left(p\right)\ln\left(p_{j}\right)dp.$$

So we have

(24) $$\begin{array}{cc} I^{C_{j}}\approx \frac{1}{2}\ln\left(\frac{N}{2\pi e}\right) & +\frac{1}{2}\int_{\Sigma_{d}}\rho \left(p\right)\ln\left(p_{j}\right)dp\\ \; & -\int_{0}^{1}\sigma \left(q_{j}\right)\ln\sigma \left(q_{j}\right)dq_{j}-\frac{1}{2}\int_{0}^{1}\sigma \left(q_{j}\right)\ln\left[q_{j}\left(1-q_{j}\right)\right]dq_{j}. \end{array}$$

Summing over all the Carols, we obtain

(25) $$\begin{array}{cc} \sum_{j=1}^{d}I^{C_{j}}\approx \frac{d}{2}\ln\left(\frac{N}{2\pi e}\right) & +\frac{1}{2}\int_{\Sigma_{d}}\rho \left(p\right)\ln\left(p_{1}\cdots p_{d}\right)dp\\ \; & -d\int_{0}^{1}\sigma \left(q\right)\ln\sigma \left(q\right)dq-\frac{d}{2}\int_{0}^{1}\sigma \left(q\right)\ln\left[q\left(1-q\right)\right]dq. \end{array}$$

Combining this result with Equation (18) and the definition (9) gives us

(26) $$\begin{array}{cc} I\approx \left(d-\frac{1}{2}\right)\ln\left(\frac{N}{2\pi e}\right) & -\int_{\Sigma_{d}}\rho \left(p\right)\ln\rho \left(p\right)dp\\ \; & -d\int_{0}^{1}\sigma \left(q\right)\ln\sigma \left(q\right)dq-\frac{d}{2}\int_{0}^{1}\sigma \left(q\right)\ln\left[q\left(1-q\right)\right]dq. \end{array}$$

Finally, we subtract $(d-\frac{1}{2})\ln N$ to obtain $\tilde{I}$ as defined in Equation (10):

(27) $$\begin{array}{cc} \tilde{I}=\left(d-\frac{1}{2}\right)\ln\left(\frac{1}{2\pi e}\right) & -\int_{\Sigma_{d}}\rho \left(p\right)\ln\rho \left(p\right)dp\\ \; & -d\int_{0}^{1}\sigma \left(q\right)\ln\sigma \left(q\right)dq-\frac{d}{2}\int_{0}^{1}\sigma \left(q\right)\ln\left[q\left(1-q\right)\right]dq. \end{array}$$

This last expression is exact, because the limit N→∞ that is part of the definition of $\tilde{I}$ eliminates the errors we have made in our approximations.

Our expression for $\tilde{I}$ depends on ρ only in the second term. So the function ρ(p) that maximizes $\tilde{I}$ is the one that maximizes

(28) $$-\int_{\Sigma_{d}}\rho \left(p\right)\ln\rho \left(p\right)dp,$$

subject, of course, to the condition that $\int_{\Sigma_{d}}\rho \left(p\right)dp=1$. The solution to this problem is well known: the integral in Equation (28) is uniquely maximized by the *uniform* distribution over the probability simplex. (This follows from the strict concavity of the function −xlnx for x>0). Recall that this is the outcome we were aiming for. So we have indeed identified an information-optimization problem that has the uniform distribution as its solution.

We can also easily find the optimal function σ(q). According to Equation (27), it is the function that maximizes the quantity

(29) $$-\int_{0}^{1}\left\{\sigma \ln\sigma +\frac{1}{2}\sigma \ln\left[q\left(1-q\right)\right]\right\}dq$$

subject to the condition $\int_{0}^{1}\sigma \left(q\right)dq=1$. We find this function by introducing a Lagrange multiplier λ and considering the quantity

(30) $$S=-\int_{0}^{1}\left\{\sigma \ln\sigma +\frac{1}{2}\sigma \ln\left[q\left(1-q\right)\right]+\lambda \sigma \right\}dq.$$

Computing the variation δS and setting it equal to zero, we find that

(31) $$\sigma \left(q\right)=\frac{A}{\sqrt{q(1-q)}}$$

for some constant *A*. We normalize σ by setting *A* equal to 1/π:

(32) $$\sigma \left(q\right)=\frac{1}{\pi \sqrt{q(1-q)}},$$

thereby arriving at a distribution we have seen before, in Equation (5). So, while the optimal ρ(p) is the uniform distribution characteristic of complex-amplitude quantum theory, the optimal σ(q) is the one arising naturally in a two-dimension real-amplitude version of quantum theory.

It is worth highlighting the crucial cancellation that made the uniform distribution the optimal choice for ρ(p): the integral over p in our expression (25) for $\sum_{j}I^{C_{j}}$ cancelled the last term in our expression (18) for $I^{B}$. It was this term that favored a distribution more heavily weighted toward the edges of the probability simplex, leading to the distribution (4).

Let us try to get an intuitive understanding of this cancellation. The reason the term $-\frac{1}{2}\int \rho \left(p\right)\ln\left(p_{1}\cdots p_{d}\right)dp$ appears in our expression for $I^{B}$ is that the statistical fluctuations are smaller near the edges of the probability simplex than near the center, in the sense that the observed frequencies of occurrence of the outcomes will typically deviate less from the outcomes’ probabilities. For the purpose of sending information to Bob, this fact makes it desirable to favor the parts of the simplex that are close to the edges. On the other hand, the amount of information Carol-*j* obtains includes a term proportional to $\ln n_{j}$, reflecting the fact that she gains more information if Bob sends her more particles to measure. The term $+\frac{1}{2}\int \rho \left(p\right)\ln\left(p_{1}\cdots p_{d}\right)dp$ appearing in Equation (25) results from a kind of compromise among all the Carols; it favors values of p such that Bob sends a respectable number of particles to each of the Carols, that is, values lying close to the center of the probability simplex. It is the competition between these two tendencies—that is, (i) the edge preference that is beneficial for communicating to Bob and (ii) the center preference that is beneficial for communicating to the Carols—that finally leads to the optimality of the uniform distribution.

## 4. Does This Information Optimization Appear in Quantum Theory?

In looking for a quantum mechanical realization of the information-optimizing scenario we imagined in Section 2 and Section 3, let us begin by assuming Alice is sending Bob a beam of *d*-dimensional particles, each prepared in the same pure state $|\psi_{A}\rangle$. Bob performs on these particles a complete orthogonal measurement $\left(\Pi_{1},\ldots ,\Pi_{d}\right)$, the probabilities of the outcomes being

(33) $$p=\left(p_{1},\ldots ,p_{d}\right)=(\langle \psi_{A}|\Pi_{1}|\psi_{A}\rangle ,\ldots ,\langle \psi_{A}|\Pi_{d}|\psi_{A}\rangle ).$$

So far, so good—but what, then, are the Carols doing? Here we consider two possibilities.

1. *Carol-j tests for another pure state*

Let us make the standard assumption that if a particle yields the *j*th outcome of Bob’s measurement—and is therefore sent to Carol-*j*—it has been collapsed into the pure state picked out by $\Pi_{j}$. Suppose that Carol-*j*’s measurement is a test for some pure state $|\psi_{C_{j}}\rangle$ unrelated to $\Pi_{j}$, and $q_{j}$ is the probability that a particle sent by Bob will pass the test. Then

(34) $$q_{j}=\langle \psi_{C_{j}}|\Pi_{j}|\psi_{C_{j}}\rangle .$$

This is a perfectly reasonable quantum mechanical scenario that one can easily imagine setting up in a lab.

For this experiment, there are quantum-mechanically natural prior measures that Bob and the Carols could use. Are they the ones that maximize the quantity $\tilde{I}$? For Bob, the answer is yes: assuming a uniform distribution of Alice’s state |ψ〉 over the sphere of pure states, we know that Bob’s prior measure for the ordered set p is the uniform measure over $\Sigma_{d}$, which, as we have seen, is the optimal measure for maximizing $\tilde{I}$.

However, for the Carols, the answer is no. If we assume Carol-*j* is initially completely ignorant of the state picked out by Bob’s projector $\Pi_{j}$, then if she were performing a *complete* orthogonal measurement, her prior measure on the (d−1)-dimensional probability simplex would be the uniform measure. Since she is not performing a complete measurement but is only distinguishing between $|\psi_{C_{j}}\rangle$ and the (d−1)-dimensional subspace orthogonal to $|\psi_{C_{j}}\rangle$, the probability distribution $\sigma (q_{j})$ she should use is the marginal distribution obtained by integrating the uniform distribution over the probabilities associated with the potential outcomes that she is not distinguishing. One finds that the result of this marginalization is

(35) $$\sigma \left(q_{j}\right)=\left(d-1\right){\left(1-q_{j}\right)}^{d-2}.$$

(Smaller values of $q_{j}$ are favored—for values of *d* greater than 2—because they leave a larger volume of $\Sigma_{d}$ available for the other probabilities. The exponent d−2 appears because, with $q_{j}$ fixed, the other probabilities occupy a space of just d−2 dimensions). This is very different from the information-optimizing distribution of Equation (32). So this example does not constitute a realization of our information-optimizing scenario, even though Bob’s ρ(p) is the one that maximizes $\tilde{I}$.

2. *Carol-j measures a “phase rebit”*

In the preceding example, we were imagining Carol acquiring information that was created by Bob’s measurement. We now consider the possibility that she is acquiring information sent by Alice but left unmeasured by Bob.

For each of the pure states distinguished by Bob’s measurement, let us choose a definite state vector $|\psi_{B_{j}}\rangle$ to represent this state, so that $\Pi_{j}=|\psi_{B_{j}}\rangle \langle \psi_{B_{j}}|$. (Our choice here is simply a matter of choosing a definite overall phase for the vector). Bob’s measurement is sensitive to part of the information contained in $|\psi_{A}\rangle$ but not to all of it. Specifically, it is sensitive to the magnitudes of the complex numbers $\langle \psi_{B_{j}}|\psi_{A}\rangle$—the squares of these magnitudes are the probabilities of Bob’s outcomes—but it is not sensitive to the *phases* of these numbers.

Let us now pretend, contrary to the actual laws of quantum theory, that these phases are preserved in the particles Bob sends to the Carols and that the Carols can gain access to them. Focusing on Carol-*j*, let us represent the phase of $\langle \psi_{B_{j}}|\psi_{A}\rangle$ as a unit vector in the complex plane. Moreover, let us treat this complex plane as the state space of a *rebit*, that is, a binary quantum object in real-vector-space quantum theory. Then Carol-*j* can make an orthogonal measurement that distinguishes, say, the real axis from the imaginary axis. The probabilities of Carol-*j*’s outcomes would then be $\cos^{2}\phi_{j}$ and $\sin^{2}\phi_{j}$, where $\phi_{j}$ is the phase of $\langle \psi_{B_{j}}|\psi_{A}\rangle$. If, as we have been assuming, Alice’s state vector $|\psi_{A}\rangle$ is initially uniformly distributed over the unit sphere in Hilbert space, then $\phi_{j}$ is uniformly distributed over the interval $0\leq \phi_{j}<2\pi$. By the same argument we used in our polarization example in the Introduction, this makes Carol-*j*’s prior distribution $\sigma (q_{j})$ equal to

(36) $$\sigma \left(q_{j}\right)=\frac{1}{\pi \sqrt{q_{j}\left(1-q_{j}\right)}},$$

which is the distribution that maximizes $\tilde{I}$.

So this example agrees perfectly with our information-optimizing scenario. It suffers, though, from the flaw that it cannot be carried out in real life.

### An Actual Quantum Mechanical Experiment

Of course phases *can* be measured in the context of an interference experiment. We now show how the preceding, impossible scenario can be turned into an experiment allowed by quantum theory.

We begin by separating the vector $|\psi_{A}\rangle$ into a “magnitude part” and a “phase part.” Using the notation introduced in the preceding example, we write

(37) $$\langle \psi_{B_{j}}|\psi_{A}\rangle =e^{i\phi_{j}}\left|\langle \psi_{B_{j}}|\psi_{A}\rangle \right|.$$

Let us now introduce a state vector $|{\tilde{\psi }}_{A}\rangle$ and a unitary transformation Φ defined as follows:

(38) $$|{\tilde{\psi }}_{A}\rangle =\sum_{j}|\psi_{B_{j}}\rangle |\langle \psi_{B_{j}}|\psi_{A}\rangle |$$

and

(39) $$\Phi =\sum_{k}e^{i\phi_{k}}|\psi_{B_{k}}\rangle \langle \psi_{B_{k}}|.$$

One can check that

(40) $$|\psi_{A}\rangle =\Phi |{\tilde{\psi }}_{A}\rangle .$$

So Alice could prepare the state $|\psi_{A}\rangle$ by first preparing the state $|{\tilde{\psi }}_{A}\rangle$ and then applying the transformation Φ.

**Figure 1:**
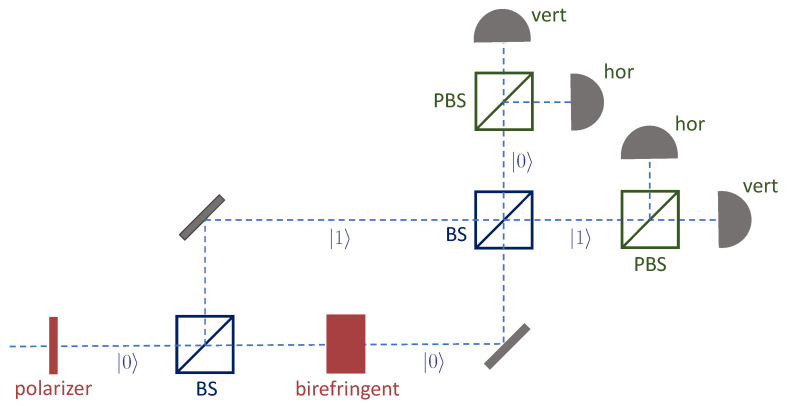
An interference experiment in which information is transferred optimally from sender to receivers. The sender (Alice) controls the linearly polarizing filter and the birefringent medium; the preferred axes of this medium are horizontal and vertical. The receivers (Bob, Carol-1, and Carol-2) have set up the beam splitters (BS), the polarizing beam splitters (PBS), and the detectors. The polarizing beam splitters have the same preferred axes as the birefringent medium.

Our interference experiment will be set up as a modified Mach–Zehnder interferometer. In this experiment, in addition to the *d*-dimensional quantum system to be prepared by Alice, there is a binary degree of freedom consisting of two possible paths that the particles might take. The path we label |0〉 consists of the particles’ initial path together with the continuation of this path via transmissions (not reflections) through two symmetric beam splitters. The path we label |1〉 is the path that begins with reflection at the first beam splitter and continues with transmission through the second. The layout is illustrated in Figure 1 for the case in which the main quantum system is photon polarization, for which *d* has the value 2.

Now, instead of having Alice prepare the state $|\psi_{A}\rangle$ at the outset, we have her prepare the state $|{\tilde{\psi }}_{A}\rangle$ initially and then apply the unitary transformation Φ only in path |0〉 just after the first beam splitter. Thus the state $|\psi_{A}\rangle$ is prepared only in that path. Then, in each of the two paths emerging from the second beam splitter, Bob performs his complete orthogonal measurement. Since he performs this measurement in two places, there are a total of 2d possible outcomes of the experiment for each incoming particle.

To compute the probabilities of the outcomes, we introduce a unitary transformation *V* representing the action of each beam splitter:

(41) $$V=\frac{1}{\sqrt{2}}\left(\begin{array}{cc} 1 & i\\ i & 1 \end{array}\right).$$

We now keep track of the effects of the three successive transformations—the first beam splitter, the application of Φ in path |0〉, and the second beam splitter—to obtain the state |Ψ〉 that will be measured by Bob. Here we take the main quantum system to be the first factor in each tensor product and the binary path variable to be the second factor.

(42) $$|\Psi \rangle =\left(I⊗V\right)(\Phi ⊗|0\rangle \langle 0|+I⊗|1\rangle \langle 1|)\left(I⊗V\right)(|{\tilde{\psi }}_{A}\rangle ⊗|0\rangle ).$$

Plugging in the definitions of $|{\tilde{\psi }}_{A}\rangle$, *V*, and Φ, we find that

(43) $$|\Psi \rangle =\frac{1}{2}\sum_{j}|\langle \psi_{B_{j}}|\psi_{A}\rangle \left|\cdot \right|\psi_{B_{j}}\rangle ⊗\left[\left(e^{i\phi_{j}}-1\right)|0\rangle +i\left(e^{i\phi_{j}}+1\right)|1\rangle \right].$$

From this we can compute the probabilities of the 2d possible outcomes of the experiment.

(44) $$\begin{array}{cc} \; & (\mathrm{probability}\;\mathrm{of}\;\mathrm{outcome}\;j\;\mathrm{in}\;\mathrm{path}\;|0\rangle )\;=|\langle \psi_{B_{j}}|\psi_{A}\rangle {|}^{2}\;\sin^{2}\left(\phi_{j}/2\right)\\ \; & (\mathrm{probability}\;\mathrm{of}\;\mathrm{outcome}\;j\;\mathrm{in}\;\mathrm{path}\;|1\rangle )\;=|\langle \psi_{B_{j}}|\psi_{A}\rangle {|}^{2}\;\cos^{2}\left(\phi_{j}/2\right) \end{array}$$

Let us think of the values $|\langle \psi_{B_{j}}|\psi_{A}\rangle {|}^{2}$ as the probabilities of the outcomes of Bob’s measurement, and the values $\sin^{2}\left(\phi_{j}/2\right)$ and $\cos^{2}\left(\phi_{j}/2\right)$ as the probabilities of the outcomes of Carol-*j*’s measurement. Then the probabilities in Equation (44), while not exactly the ones we encountered in our illegal example of the “phase rebits,” differ from those probabilities only by the factor 1/2 inside the sine and cosine. If $\phi_{j}$ is uniformly distributed, as we are assuming, then $\sin^{2}\phi_{j}$ and $\sin^{2}\left(\phi_{j}/2\right)$ both inherit the same distribution over the interval [0,1], namely, the one given by Equation (36). So in this experiment, Bob’s ρ(p) and Carol-*j*’s $\sigma (q_{j})$ both turn out to be the functions that maximize the quantity $\tilde{I}$.

Let us identify the components of the experiment associated with the roles of Alice, Bob, and the Carols. As we have already suggested, Alice controls the device that prepares the initial state $|{\tilde{\psi }}_{A}\rangle$ and the device that implements the unitary transformation Φ. To identify the Bob and Carol components, it helps to imagine each of the *d*-outcome measurements as consisting of a splitting device $S_{k}$, which merely splits the beam into *d* subbeams, and a set of *d* detectors $D_{jk}$. Here the index *k* takes the values 0 and 1 and labels the path on which the splitting device and the detectors appear. Then we can locate our participants’ portions of the apparatus as follows:
Alice:Devices associated with $|{\tilde{\psi }}_{A}\rangle$ and Φ.Bob:$S_{0}$, $S_{1}$, and all the detectors.Carol-*j*:The two beam splitters, plus detectors $D_{j0}$ and $D_{j1}$.

Note that our participants are not well separated from each other. Two of the overlaps are particularly noteworthy: (i) Alice must know in advance what measurement Bob will make, because her unitary transformation Φ needs to be diagonal in the preferred basis of that measurement; (ii) in order to make their phase measurements, the Carols must interrupt Alice’s preparation by inserting their initial beam splitter. The latter of these two overlaps is what prevents us from interpreting the experiment as a clean preparation followed by a measurement.

Thus we have imagined a genuine quantum mechanical experiment in which information is transmitted optimally from the sender (Alice) to the receivers (Bob and the Carols), but the experiment involves a basis-specific separation between the “magnitude” and “phase” components of the prepared state, and the receivers need to intervene in the middle of the preparation in order to be able, later, to extract certain features of the transmitted information.

## 5. Discussion

It is an intriguing fact that, according to the quantum theory of a system with a finite-dimensional Hilbert space, a uniform distribution over the unit sphere of pure states induces the uniform distribution over the probability simplex. This fact could conceivably help us understand why quantum theory has the structure it has, *if* we can attribute to the latter distribution some foundational significance. In this paper, we have explored the possibility that the uniform distribution over the probability simplex might have the significance of allowing information to be transmitted optimally, in some appropriate understanding of that phrase.

We have identified a specific information-optimization problem for which the uniform distribution over the simplex does emerge as part of the solution. The problem begins with a simple communication scenario between a sender, Alice, and a receiver, Bob. It continues by assuming that for each outcome of Bob’s measurement, there is a further binary measurement, and the information provided by these binary measurements is included in the tally. The solution of this problem consists of two distributions: a distribution ρ(p) over $\Sigma_{d}$, where *d* is the number of outcomes of Bob’s measurement, and a distribution σ(q) over the one-dimensional simplex $\Sigma_{2}$. The first of these is indeed the uniform distribution. The other is the distribution given in Equation (32).

It is interesting that each of these distributions, taken separately, appears naturally in quantum theory. The uniform distribution has, of course, been the focus of this paper. Meanwhile, the distribution (32), in addition to arising in the linear polarization problem as discussed in the Introduction, arises also in the simplest possible interference experiment. Consider a basic Mach–Zehnder interferometer with a simple phase shifter in one of the two paths. (This is a special case of the interference experiment described in Section 4, with *d* having the value 1). Let *q* and 1−q be the probabilities of the two possible paths emerging from the second beam splitter. If the phase ϕ that is added by the phase shifter is uniformly distributed over the interval 0≤ϕ<2π, then *q* is distributed according to the function

(45) $$\sigma \left(q\right)=\frac{1}{\pi \sqrt{q(1-q)}},$$

which is the optimal σ(q) for our communication problem.

The interference experiment we have presented in Section 4 brings these two optimal functions, ρ(p) and σ(q), together by embedding a basic prepare-and-measure experiment into a Mach–Zehnder interferometer. While this experiment does indeed exhibit optimal information transfer from the sender to the receivers, it does so at the cost of requiring the receivers to intervene in the middle of the preparation procedure. So the experiment seems a bit artificial, just as the polarization experiment we described in the Introduction feels artificial in that it requires ignoring the possibility of circular or elliptical polarization.

Essentially, what the additional binary measurements in our problem are doing, in the quantum context, is to turn the real and imaginary parts of each quantum amplitude into two dimensions of a real vector space. The interference experiment of Section 4 is basically a scheme for making standard quantum theory look like real-vector-space quantum theory—the interference between the two paths provides a way of accessing those real and imaginary components. As we have noted in the Introduction, it was already known that in real-vector-space quantum theory, the transfer of information from Alice to Bob (with no extra measurements after Bob’s measurement) is optimal. So we are effectively observing the same result again in this paper. What is different here is that our framing of the communication problem, which separates Bob from the Carols, allows us to see the how the *uniform* distribution over $\Sigma_{d}$, which is characteristic of standard, complex-amplitude quantum theory, emerges from the extremization.

Still, though, this line of thinking—that is, asking about optimal information transfer under the assumption that the state being transmitted is pure—continues to single out the real-vector-space theory as special in a certain sense. It is in this theory that a complete orthogonal measurement accesses every dimension of the manifold of pure states (as opposed to the complex theory, in which such a measurement misses the phase dimensions), and it does so in a way that allows information to be conveyed optimally. One can imagine reconstructing quantum theory by starting with an information-optimizing theory but then imposing a rule that forbids measurement of the “phase rebits.” That is to say, one could start with Bob and the Carols, but then assume that no one can actually carry out the Carols’ measurements. Indeed, something along these lines can be seen in Goyal’s 2010 reconstruction of quantum theory, in which one assumes the existence of a binary degree of freedom that is an essential part of the theory but is inaccessible [14].

Very recently, theoretical and experimental advances have shown quite clearly that, within the standard framework of quantum axioms, real-vector-space quantum theory can be ruled out [15,16,17,18], and yet we have known since the work of Stueckelberg that it is possible to express quantum theory purely in terms of a real Hilbert space [19]. These two facts are not inconsistent, because Stueckelberg’s formulation does not respect the usual tensor-product rule for describing composite systems. The work presented in the present paper gives us reason not to set real-vector-space theories entirely aside. 

## Data Availability

Data sharing not applicable.
